# A new predictive tool consolidating CURB-65 with procalcitonin and albumin to assess short-term mortality in hospitalized elderly patients with infectious disease: A retrospective study of a patient cohort

**DOI:** 10.1097/MD.0000000000031614

**Published:** 2022-11-18

**Authors:** Toshihiro Higashikawa, Tomohiko Ito, Takuro Mizuno, Keiichirou Ishigami, Kengo Kuroki, Naoto Maekawa, Daisuke Usuda, Shinichiro Nakao, Kazu Hamada, Susumu Takagi, Nao Terada, Kento Takeshima, Shinya Yamada, Ryusho Sangen, Toshihide Izumida, Jun Kiyosawa, Atsushi Saito, Masaharu Iguchi, Hiroyuki Wato, Takeshi Nakahashi, Yuji Kasamaki, Akihiro Fukuda, Tsugiyasu Kanda, Masashi Okuro

**Affiliations:** a Kanazawa Medical University Himi Municipal Hospital, Kurakawa, Himi, Toyama, Japan; b Department of Geriatric Medicine, Kanazawa Medical University, Uchinada, Kahoku-gun, Ishikawa, Japan; c Department of Emergency and Critical Care Medicine, Juntendo University Nerima Hospital, Takanodai, Nerima-ku, Tokyo, Japan.

**Keywords:** albumin, CURB-65, hospitalized elderly patients, procalcitonin

## Abstract

Hospitalized elderly patients are often at risk of life-threatening infectious diseases such as pneumonia and urinary tract infection, thus diagnostic tools for bacterial infections are demanded. We developed a new predictive tool consolidating modified CURB-65, procalcitonin (PCT) and albumin (Alb).

**Method:**

This is a retrospective study. Modified CURB-65 (mCURB-65) score, PCT, Alb, and various cardiovascular/respiratory/renal functions were measured. Survival analyses were conducted to assess 30-days mortality of elderly patients using mCURB-65 score, PCT and Alb. The consolidated scores were compared with the number of patients died.

**Results:**

There were 445 elderly patients included. Kaplan–Meier survival curves showed significant differences between the high and low groups of mCURB-65, PCT and Alb (log-rank test, *P* < .001). Cox proportional regression showed that the hazard ratios (95% confidence intervals) for high mCURB-65, high Alb, and high PCT were all significant, 1.95 (1.24–3.05), 0.50 (0.32–0.77), and 2.09 (1.32–3.31), respectively. The consolidated scores showed tendency of increase with proportion of the number of patients died.

**Conclusions:**

The consolidated score consisted of mCURB-65, PCT and Alb can be a useful tool to predict short-term mortality of the hospitalized elderly patients with infectious disease.

## 1. Introduction

Elderly people are at risk of developing diseases of various internal organs, such as heart failure, renal failure and chronic respiratory disease, pneumonia, urinary tract infection, inflammation of the gallbladder, and tend to have poor frail and nutritional status.^[1–4]^ The source of infection tends to spread throughout the body of the patients and often extends to multiple organs rather than a single organ. Some diseases such as pneumonia and urinary tract infections can become lethal, thus some predictive tools to assess disease severity and short-term mortality of the elderly patients are of paramount importance. For disease severity assessment, CURB-65 score was first proposed by Lim et al,^[5]^ and has been widely applied to assess the prognosis of community-acquired pneumonia.^[6–14]^ CURB-65 does not include nutritional or personal care assistance and is a convenient indicator which stands for 5 easily measurable parameters (confusion, raised urea, tachypnea, hypotension and age ≥ 65). The CURB-65 was also applied to predict 30 day mortality in patients admitted to a general internal medicine ward.^[15]^ Several studies have also suggested biochemical parameters such as procalcitonin (PCT)^[7,8,11,16–19]^ and albumin (Alb)^[6,10,12,17,20–22]^ as valuable indices to predict health status and quality of life of hospitalized elderly patients to a certain extent. Other than CURB-65, qSOFA also exists as an index of organ damage.^[23]^ Because various scoring systems have been available which might interfere decision to select in the real-world clinical settings, optimal scoring system has been demanded. However, little information is available concerning convenient methods to comprehensively assess the short-term mortality of the hospitalized elderly patients who are at risk for death.

The objective of the present study was to investigate association of CUBR-65 with several biochemical parameters and to establish a new predictive tool to comprehensively assess severity of disease and prognosis of Japanese elderly patients admitted to hospital.

## 2. Materials and Methods

The study was approved by the Clinical Research Ethics Committee of the Kanazawa Medical University Himi Municipal Hospital. The Committee may grant a waiver of informed consent in certain instances.

Herein, we performed a retrospective study to investigate the association of modified CURB-65, PCT and Alb in elderly patients admitted to the hospital. This study included elderly patients admitted to the Kanazawa Medical University Himi Municipal Hospital from 2013 to 2016. The patients who affected bacterial (not viral) infection, followed by measurement of PCT, Alb, C-reactive protein, hemoglobin, total billirubin and blood urea nitrogen levels in blood samples when admitted to the hospital were included. The patients underwent medication of antiboitics. The patients aged < 65 years, no use of antibiotics, not infectious disease and out-hospital diagnoses were excluded. Demographic and baseline characteristics were collected from the patients’ medical records. Modified CURB-65 (mCURB-65) score was measured by collecting parameters when admitted to the hospital (confusion, blood urea nitrogen > 21 mg/dL, percutaneous oxygen saturation (SpO_2_) < 90%, systolic blood pressure < 90 mm Hg and/or diastolic blood pressure ≤ 60 mm Hg, and age ≥ 65. The “modified” is the mean that criteria of SpO_2_ < 90% was applied instead of respiratory rate ≥ 30/min, because a convenient saturation monitor has been widely used in Japan. PCT concentration of < 0.5 ng/mL was defined as PCT-negative, and a concentration of ≥ 0.5 ng/mL was defined as PCT-positive. Accordingly, Alb concentration of < 2.5 g/dL was defined as Alb-deficient, and a concentration of ≥ 2.5 g/dL was defined as Alb-sufficient.

A *t* test and chi-square test were performed to compare significant differences between the 2 groups (survived and died). 5X2 cross-table chi-square tests were also performed to evaluate the relationship between the scores of mCURB-65 and negative/positive PCT and deficient/sufficient Alb.

Survival analyses were performed to assess the effect of high mCURB-65 score, high PCT and low Alb as risk factors for mortality in hospitalized elderly patients. We created Kaplan–Meier estimated survival curves where the observation period was defined as 30 days from the blood sample collection, and the event was defined as death occurred during the 30 days period. Significant differences between the 2 groups were evaluated by log-rank test, and the overall difference at 30 days between the 2 groups was evaluated by the hazard ratio and 95% confidence interval by applying a Cox proportional hazards model, with gender and age as covariates. Furthermore, consolidated CURB-65 was defined as mCURB-65 plus PCT score (0: <0.5 ng/mL, 1: ≥0.5 ng/mL) plus Alb score (0: ≥2.5 g/dL, 1: <2.5 g/dL), and its association with proportion of the number of patients died on each score was evaluated.

All statistical analyses were 2-tailed, and statistical significance was set at *P* < .05. Data were analyzed using the freely available EZR (Easy R) software (Saitama Medical Center, Jichi Medical University, Saitama, Japan).^[24]^

## 3. Results

**Figure 1.**
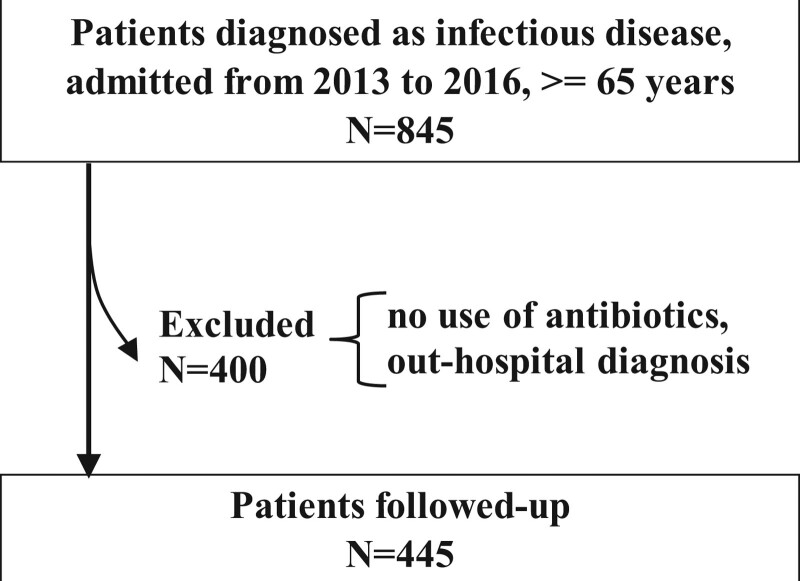
Flow chart for the study.

The number of patients included in the present study is 445 as shown in Figure 1.

The population includes 273 males and 172 females. The mean ± standard deviation of the age of the population is 82.5 ± 7.9 years. The 85 patients died within the 30 day observation period, and 360 patients survived. None of the patients discharge during the observation period. The demographics and physiological variables of the patient’s background are summarized in Table 1.

**Table 1:** Demographics and physiological variables of patients background.

	Survived (N = 360)	Died (N = 85)	※P value
Gender (male/female)	213/147	60/25	.0518
Age (mean ± SD)	83.78 ± 7.83	81.35 ± 8.02	.1345
mCURB-65 (3–5/1–2)	116/244	52/33	<.001
PCT (>=0.5/<0.5 ng/mL)	127/233	55/30	<.001
ALB g/dL	2.90 ± 0.68	2.31 ± 0.52	<.001
ALB (>=2.5/<2.5 g/dL)	263/97	35/50	<.001
CRP (mg/dL)	7.88 ± 6.91	10.79 ± 7.60	<.001
ADL (JA/BC)	129/231	28/57	.6157
Hemoglobin g/dL	11.52 ± 2.27	11.14 ± 2.15	.1544
Diastolic blood pressure (mm Hg)	72.10 ± 18.83	69.70 ± 18.89	.2959
Systolic blood pressure (mm Hg)	127.49 ± 27.09	123.82 ± 30.03	.2741
Heart rate (bpm)	91.14 ± 35.74	94.57 ± 22.53	.4041
Body temperature (deg)	37.44 ± 1.17	37.24 ± 1.10	.1711
SpO2 (<90%/>=90%)	109/251	49/36	<.001
Impaired consciousness (Posi/Nega)	106/254	32/53	.1414
T-BIL (mg/dL)	0.83 ± 0.75	1.27 ± 2.48	.006
BUN (mg/dL)	26.16 ± 20.01	36.23 ± 26.57	<.001
CRE (mg/dL)	1.45 ± 1.87	1.76 ± 1.90	.1739
Serum Na + (mEq/L)	136.74 ± 5.73	137.62 ± 7.06	.2253
Serum K + (mEq/L)	4.25 ± 1.90	4.09 ± 0.92	.4457
Serum Cl- (mEq/L)	101.91 ± 5.79	102.38 ± 7.38	.5264

ADL = activity of daily living, ALB = albumin, BUN = blood urea nitrogen, CRE = creatinine, CRP = c-reactive protein, PCT = procalcitonin, SpO2 = percutaneous oxygen saturation, T-BIL = total billirubin.

※ Chi-square test (Gender, CURB65, PCT, ALB, ADL), *t* test (others).

Regarding mCURB-65, PCT, Alb, and SpO2, association of these variables with groups (survived and died) are significantly dependent (*P* < .001). Regarding CRP, T-BIL and BUN, significant differences are observed between the groups (*P* < .001). On the other hand, gender, age, ADL, hemoglobin, blood pressure, heart rate, body temperature, impaired consciousness, creatinine and serum electrolytes, no significant differences are found between the groups.

The association of procalcitonin level (high/low) with mCURB-65 score show significant difference (Table 2; df = 4, *P* < .001).

**Table 2:** Association of procalcitonin with mCURB-65 score.

	mCURB-65					
	1	2	3	4	5	Total
Procalcitonin (–)	68	124	58	13	0	263
Procalcitonin (+)	28	57	54	38	5	182
Total	96	181	112	51	5	445
						*P* < .001 by Chi-square test

mCURB = modified confusion, uremia, respiratory rate, blood pressure.

The association of albumin level (high/low) with mCURB-65 score does not show significant difference (Table 3; df = 4, *P* = .2323).

**Table 3:** Association of albumin with mCURB-65 score.

	mCURB-65					
	1	2	3	4	5	Total
Albumin (–)	25	56	45	19	2	147
Albumin (+)	71	125	67	32	3	298
Total	96	181	112	51	5	445
						*P* = .2323 by Chi-square test

mCURB = modified confusion, uremia, respiratory rate, blood pressure.

**Figure 2.**
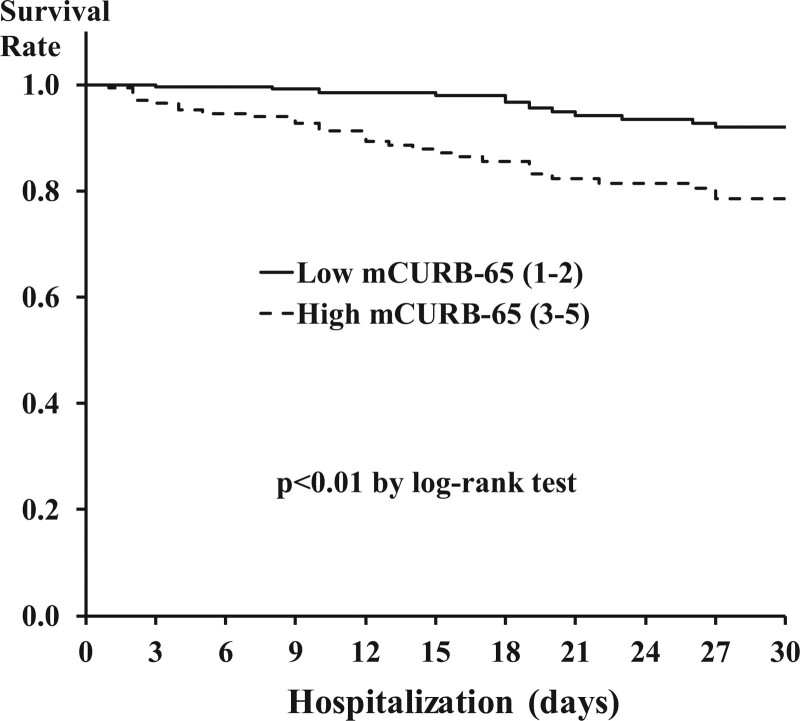
Cumulative survival rate according to low/high mCURB65 score. mCURB = modified confusion, uremia, respiratory rate, blood pressure.

The Kaplan–Meier survival analysis in the low mCURB-65 score (1–2) and high mCURB-65 score (3–5), the survival rate is significantly lower in the high mCURB-65 than low mCURB-65 group, as shown in Figure 2 (log-rank test, df = 1, *P* < .001).

**Figure 3.**
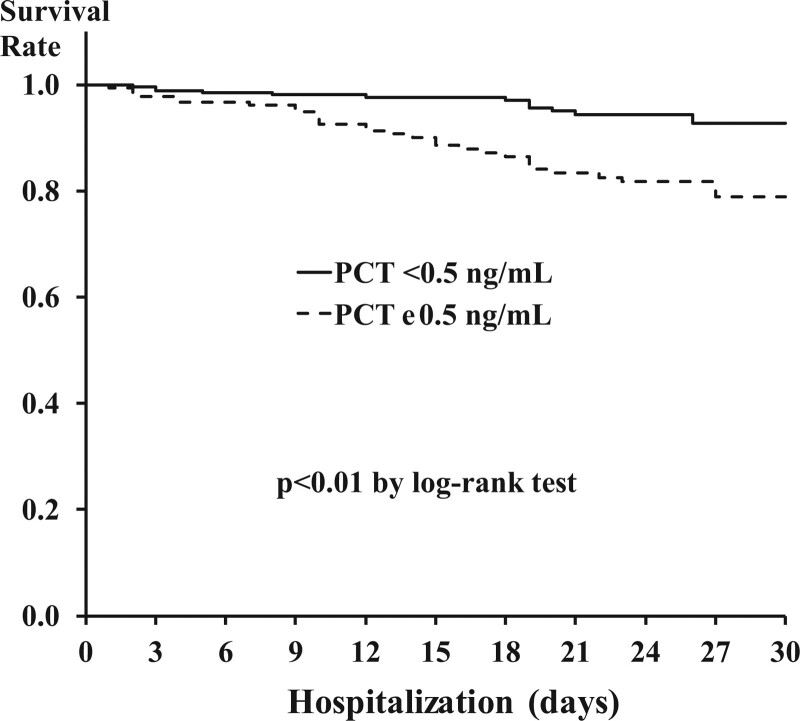
Cumulative survival rate according to low/high procalcitonin score.

The Kaplan–Meier survival curves of PCT-positive and negative groups, where the survival rate is significantly lower in the PCT-positive than negative group, as shown in Figure 3 (log-rank test, df = 1, *P* < .001).

**Figure 4.**
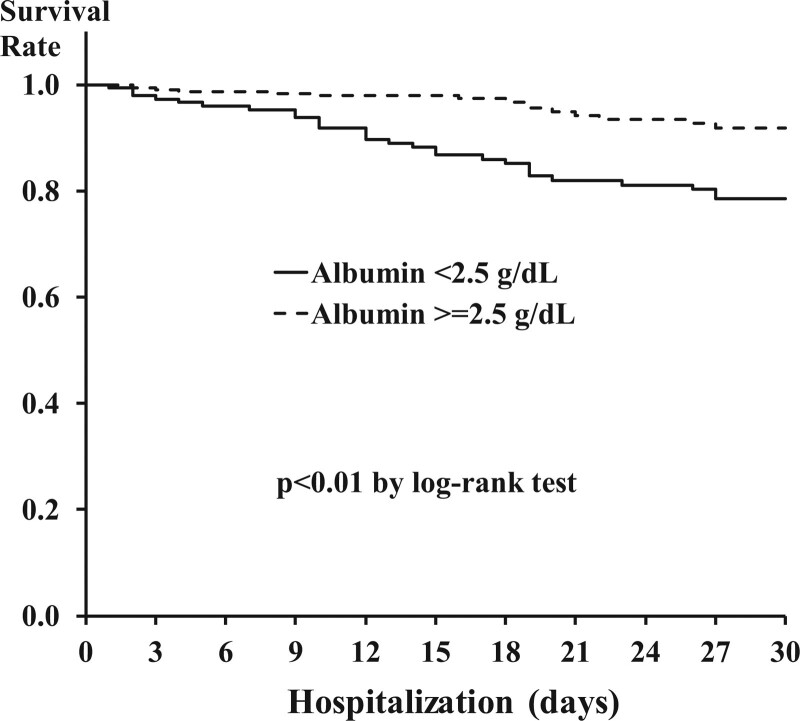
Cumulative survival rate according to low/high albumin score.

The Kaplan–Meier plots of Alb-deficient and sufficient groups, where the survival rate is significantly lower in the Alb-deficient than sufficient group, as shown in Figure 4 (log-rank test, df = 1, *P* < .001).

The age and sex-adjusted Cox regression analyses show that hazard ratios, quantified the difference between the hazard of 2 groups, show significantly high in high mCURB-65 (1.95, *P* < .001) and high PCT (2.09, *P* < .001), whereas high Alb show significantly low hazard ratio (0.50, *P* < .001), as shown in Table 4.

**Table 4:** Hazard ratio calculated by age and sex-adjusted Cox regression model.

	Hazard Ratio	Lower 95% CI	Upper 95% CI	*P* value
Age	1.01	0.98	1.04	.5640
Sex	1.11	0.68	1.81	.6761
High mCURB-65	1.95	1.24	3.05	.0016
High PCT	2.09	1.32	3.31	.0018
High Alb	0.50	0.32	0.77	.0037

Alb = albumin, CI = confidence interval, PCT = procalcitonin.

**Figure 5.**
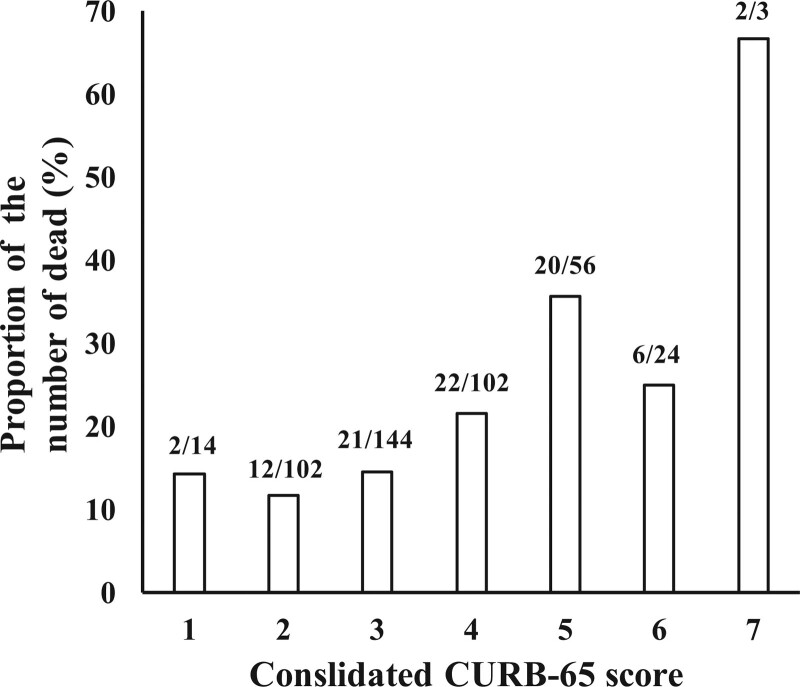
Proportion of the number of death on each consolidated CURB-65 score. CURB = confusion, uremia, respiratory rate, blood pressure.

The consolidated CURB-65 score (mCURB-65 plus PCT plus Alb; 1-7) and its association with proportion of the number of died patients on each score shows the proportion of the number of dead patients is found to increase in accordance with the increase in the score, as shown in Figure 5.

## 4. Discussion

Our results have confirmed that the status of high mCURB-65 score, high PCT and low Alb can be significant risk factors for the survival of hospitalized elderly patients with infectious disease, and consolidated CURB-65, a new consolidated scoring index (mCURB-65 score plus PCT score plus Alb score), can be a useful and convenient tool to assess severity and prognosis of the elderly patients. This study has also revealed significant associations of mCURB-65 with PCT.

Alb and PCT (Alb and PCT take several days to improve the value even with treatment) are combined and scored, it is essential that various parameters are added to the scores increase the accuracy of the diagnosis. The mCURB-65 includes confusion and blood pressure, thus it could be positively correlated with PCT as a biomarker of sepsis that is produced in inflamed organs with a rapid increase in the first phase followed by a slow rate of decrease in the second phase. On the other hand, Alb is produced in the liver with a long half-life, and a low Alb level, which reflects nutritional status, may lead to various complications, thus it could be negatively correlated with mCURB-65.

Our previous study has already confirmed that dual measurement of PCT and Alb can be a valuable tool to assess prognosis in elderly people.^[17]^ PCT enhancement was also observed in noninfectious diseases. Not only bacteria-derived substances, endotoxin, but also physiologically intracellular and extracellular-released substances triggered the production of PCTs by recognizing substance derived from bacterial pathogen-associated molecular patterns or damage associated molecular patterns, but non-physiological apoptosis of the cells results in the release of material with similar molecular patterns to enhance the production of PCT.^[25,26]^

In parallel, for the assessment of prognosis of patients with community-acquired pneumonia, combined use of CURB-65 and PCT,^[7–9,11,16,18,19]^ and combined use of CURB-65 and Alb,^[6,10,12–14,21,27]^ have been widely applied. Alb is known to be a prognostic factor for elderly people.^[20]^ Patients with malnutrition, low Alb level, could be a risk factor for mortality due to infectious diseases.^[28]^ Our previous study also suggests that Alb could be a risk factor for the development of aspiration pneumonia in elderly patients with femoral neck and trochanteric fractures.^[29]^ These studies suggest that dual measurement procedure can improve prognostic accuracy rather than single CURB-65 measurement. However, the optimal combination of these indices remains somewhat vague.

The strength of the present study is that CURB-65, including confusion, blood urea nitrogen, respiratory rate, and blood pressure, which are used for measuring severity of pneumonia, can be influenced by concomitant illness or other medications due to chronic disease, thus concomitant measurement of PCT and Alb, as well as establishment of consolidated scoring tool (cCURB-65) can be valuable in evaluating the severity of infectious disease and in improving prognostic accuracy. Recently, CURB-65 has been applied in patients with coronavirus disease 2019 in China, but its prediction accuracy of in-hospital death was still not enough.^[30]^

The present study had some limitations. First, the study population includes patients who admitted in other departments of our hospital or patients who remained in the emergency observation room. Such heterogeneous nature of the patient population could be a cause of confounding which cannot be excluded. Second, uncontrolled antibiotics use and/or nutritional therapy between the groups may serve as a potential confounding factor.

Third, due to the lack of sensitivity PCT was measured and analyzed as discrete variables and not continuous variables, which may lead to the loss of information to some extent.

## 5. Conclusion

In conclusion, a new scoring tool cCURB-65, including mCURB-65, PCT and Alb, is a simple and effective tool in assessing the disease severity and prognosis of hospitalized elderly patients with infectious disease. Despite the encouraging results, further validation would be warranted in future multicenter large prospective studies.

## Acknowledgments

We thank Yojiro Sakiyama, PhD, from Medinfo K.K. (https://statg.com) for commenting statistics and editing a draft of this manuscript.

## Author contributions

**Data curation:** Toshihiro Higashikawa, Keiichiro Ishigami.

**Formal analysis:** Toshihiro Higashikawa, Tomohiko Ito, Takuro Mizuno.

**Funding acquisition:** Toshihiro Higashikawa.

**Investigation:** Toshihiro Higashikawa, Kengo Kuroki, Naoto Maekawa, Daisuke Usuda.

**Methodology:** Toshihiro Higashikawa, Shinichiro Nakao, Kazu Hamada, Daisuke Usuda, Susumi Takagi, Nao Terada, Kento Takashima, Shinya Yamada, Ryusho Sangen, Toshihide Izmida, Jun Kiyosawa, Atsushi Saito, Masaharu Iguhchi, Hiroyuki Wato.

**Project administration:** Toshihiro Higashikawa.

**Resources:** Toshihiro Higashikawa.

**Software:** Toshihiro Higashikawa.

**Supervision:** Masashi Okuro, Takeshi Nakahashi Yuji Kamasaki, Akihiro Fukuda, Tsugiyasu Kanda.

**Validation:** Toshihiro Higashikawa.

**Visualization:** Toshihiro Higashikawa.

**Writing – original draft:** Toshihiro Higashikawa.

**Writing – review & editing:** Toshihiro Higashikawa.
